# O8.2. DURATION OF UNTREATED PSYCHOSIS (DUP) IS ASSOCIATED WITH WORSE RESPONSE TO TREATMENT IN ANTIPSYCHOTIC NAïVE FIRST EPISODE PSYCHOSIS

**DOI:** 10.1093/schbul/sby015.238

**Published:** 2018-04-01

**Authors:** Daniel Cavalcante, Bruno Ortiz, Mariane Noto, Quirino Cordeiro, Luccas Coutinho, Vanessa Ota, Sintia Belangero, Rodrigo Bressan, Ary Gadelha, Cristiano Noto

**Affiliations:** 1 Universidade Federal de São Paulo; 2 Santa Casa de Misericordia de São Paulo

## Abstract

**Background:**

Longer duration of untreated psychosis (DUP) predicts poor functional outcomes in patients with schizophrenia. The results are not so clear when addressing effects on specific dimensions of symptoms, which may be due to confounding effects of previous antipsychotic treatment. We investigated the association between DUP, specific dimensions and response to treatment in a 10-week follow up study of patients at First-Episode of Psychosis (FEP) with no previous antipsychotic use.

**Methods:**

We assessed 158 antipsychotic naïve individuals with first-episode psychosis, admitted to a psychiatric emergency service. Diagnosis was established according to the Structured Clinical Interview for DSM-IV (SCID). Symptom severity was measured with the Positive And Negative Symptoms Scale (PANSS) and the Clinical Global Impressions Scale (CGI). Functionality was assessed with the Global Assessment of Functioning Scale (GAF). All patients were treated with risperidone and reassessed after 10 weeks of treatment. For analyses, we performed non-parametric correlation tests (Spearman’s correlation).

**Results:**

At baseline, we did not find a correlation between DUP and symptom severity and functionality. After the follow-up, DUP became significantly correlated to both symptomatic and functional outcomes. DUP showed significant association with PANSS positive score (r=0.282; p=0.008), PANSS negative score (r=0.295; p=0.005), PANSS total score (r=0.258; p=0.017), CGI total (r=0.305; p=0.003) and GAF (r=-0.294; p=0.004). We also found a negative correlation between DUP and response to treatment considering 30% of reduction of PANSS’ scores (r=-0.288; p=0.027).

**Discussion:**

Our findings support that DUP does not affect the severity of illness at baseline, but modifies the response to treatment and clinical severity after 10 weeks. This finding suggests that longer exposition to psychosis might be involved in biological abnormalities that modulate the response to antipsychotics, which could mediate poor response to treatment.

